# Study on stress and deformation characteristics of existing-new two-stage cantilever retaining wall

**DOI:** 10.1371/journal.pone.0296330

**Published:** 2024-02-09

**Authors:** Xuening Ma, Yuhang Liu, Zixiang Hao, Xu Wang, Youhai Yang

**Affiliations:** School of Civil Engineering, Lanzhou Jiaotong University, Lanzhou, China; University of Sharjah, UNITED ARAB EMIRATES

## Abstract

A two-stage cantilever retaining wall is composed of two single-stage cantilever retaining walls, which are stacked up and down. The structure not only has the advantages of a single-stage retaining wall, but also compensates for the shortcomings of the height limit of the single-stage retaining wall; therefore, it has been gradually applied in projects. Based on the actual project of Zhongwei-Lanzhou Passenger Dedicated Line into Lanzhou Hub, this paper studies the influence of the construction of new cantilever retaining wall and the filling of subgrade on the deformation and earth pressure of the new cantilever wall and the existing cantilever wall by means of field test and numerical simulation. The results show that with an increase in the filling height after the new cantilever wall (upper wall), the horizontal displacement of the top of the upper and lower walls increased nonlinearly. The displacement direction of the upper wall was the filling direction, and that of the lower wall was the deviation from the filling direction. The higher the filling height, the greater is the displacement. With an increase in the filling height, the earth pressure behind the upper wall increases gradually along the wall height and decreases slightly to the bottom of the wall, which is approximately a linear distribution. The earth pressure behind the existing cantilever wall first increases along the wall height and gradually decreases after reaching a certain depth, but the earth pressure of the lower wall does not increase significantly with an increase in the filling height behind the upper wall. The slope failure mode is the overall sliding failure of the retaining wall together with the fill soil. The sliding surface passed through the lower edge of the lower wall heel and was similar to an arc shape. The stability of the two-stage cantilever retaining wall was better than that of a single-stage retaining wall. Finally, a calculation method for the overall stability and earth pressure of the existing two-stage cantilever retaining wall was proposed.

## 1. Introduction

With the rapid development of transportation infrastructure, retaining walls have been widely used in highways, railways, water conservancy, and other projects. In the application, more consideration is given to its light, flexible, mechanized construction, and full use of building materials. As one of the most commonly used light retaining walls, a cantilever retaining wall is a type of reinforced concrete thin-walled retaining structure rigidly connected by a vertical arm and wall bottom plate. Its structure is simple, lightweight, and can be prefabricated and assembled onsite. It has low requirements on the bearing capacity of the foundation, and also relies on the weight of the filler behind the wall to maintain the stability of the structure; therefore, it has been more widely developed and applied [1–5].

With the promotion of the Belt and Road Initiative and the expansion of transportation demand, the reconstruction of existing lines and new projects adjacent to existing lines are also increasing annually. This type of project inevitably involves a subgrade widening project. For some high embankments of the existing lines, a cantilever retaining wall is often used to close the slope. When subsequent new lines or reconstruction and expansion projects are close to the laying of such embankments, owing to the limitation of topographic conditions, a new cantilever retaining wall can be added above the existing cantilever wall to fill the new embankment directly on the existing embankment slope. This type of retaining structure is collectively referred to as an existing two-stage cantilever retaining wall. It not only has the characteristics of a single-stage cantilever retaining wall but also has the advantage of a gravity retaining wall, which is more conducive to the overall stability of the retaining wall.

In recent years, engineering and academic circles have paid more attention to cantilever retaining walls [6–17], but there have been few studies on multi-stage cantilever retaining walls. Liang [18] simulated construction conditions using a numerical analysis method and analyzed the deformation and stress characteristics of a two-stage stacked cantilever retaining structure. Based on the plastic limit analysis theory of soil, Wang [19] studied the earth pressure analysis model of a two-stage cantilever retaining wall and provided a theoretical calculation formula for the earth pressure. To investigate multi-step cantilever retaining wall mechanical behaviours fully to support practical analysis and design of the novel wall, a series of laboratory model tests is conducted for three-step cantilever walls retaining cohesionless filling under various strip surcharges on the top of the backfill to study lateral earth pressure distribution[20]. Through theoretical and case analyses, Liang [21] showed that the base stress distribution of a two-stage cantilever retaining wall is more reasonable and safer than that of a single-stage cantilever retaining wall. Liu [22], through the field test results of the earth pressure of a multi-stage retaining wall on a high-fill slope, showed that the horizontal earth pressure behind the multi-stage retaining wall is nonlinearly distributed along the wall height with the upper filling height, and the design theory of the single-stage retaining wall cannot be simply applied to a multi-stage retaining wall. Aiming at the multilevel retaining wall project of a highway, Fan [23] studied the earth pressure and deformation of a multilevel retaining wall by means of a field prototype test, theoretical analysis, and numerical simulation. He [24] and Mao [25] studied the dynamic characteristics of a two-stage cantilever retaining wall under a horizontal earthquake, using a shaking table model test. Kasif [26] used the 3D FEM in time domain to investigate the effects of various configurations on seismic response of the cantilever retaining walls considering soil-structure interaction. Kasif [27] adopt the FEM to study the effects of different ground motions, backfill-structure interaction and soil-structure interaction on dynamic response of a box culvert wing wall. To determine seismic permanent displacement of a new type of assembled multi-step cantilever retaining walls, He [28] adopt the upper bound limit analysis and pseudo-static method to derive calculation formulas of seismic horizontal permanent displacement at the top of each step of the wall including two, three, and four steps. Based on Coulomb ’s earth pressure theory, Jia [29] calculated the seismic active earth pressure of the upper and lower walls of a two-stage cantilever retaining wall under the graded wall back theory and overall wall back theory under different peak accelerations by combining the Mononobe-Okabe method. In terms of stability analysis, previous studies have discussed the stability of single-stage cantilever retaining walls [30], but there are few calculation methods involving the overall stability of multistage retaining wall slope [31, 32].

In summary, a two-stage cantilever retaining wall was applied for slope reinforcement. However, the design and calculation method of the two-stage cantilever retaining wall is not mature, and there are no corresponding design regulations in the current design code. The stress and deformation properties need to be investigated further. Therefore, this study relies on the Zhongwei-Lanzhou passenger dedicated line to introduce the Lanzhou hub supporting project and builds a new cantilever retaining wall on the slope of the existing embankment cantilever retaining wall to realize the widening roadbed. These two components constitute a two-stage cantilever retaining wall structure. The field test and numerical simulation method were used to study the development law of soil pressure, deformation, and overall stability during the construction and filling of the new cantilever retaining wall, and the design and calculation method of the two-stage cantilever retaining wall were established. The research results have important theoretical value and engineering significance for the decision-making, design, and construction of existing line reconstructions and adjacent existing line construction projects.

## 2. Project profile

### 2.1 Design overview

**Fig 1:**
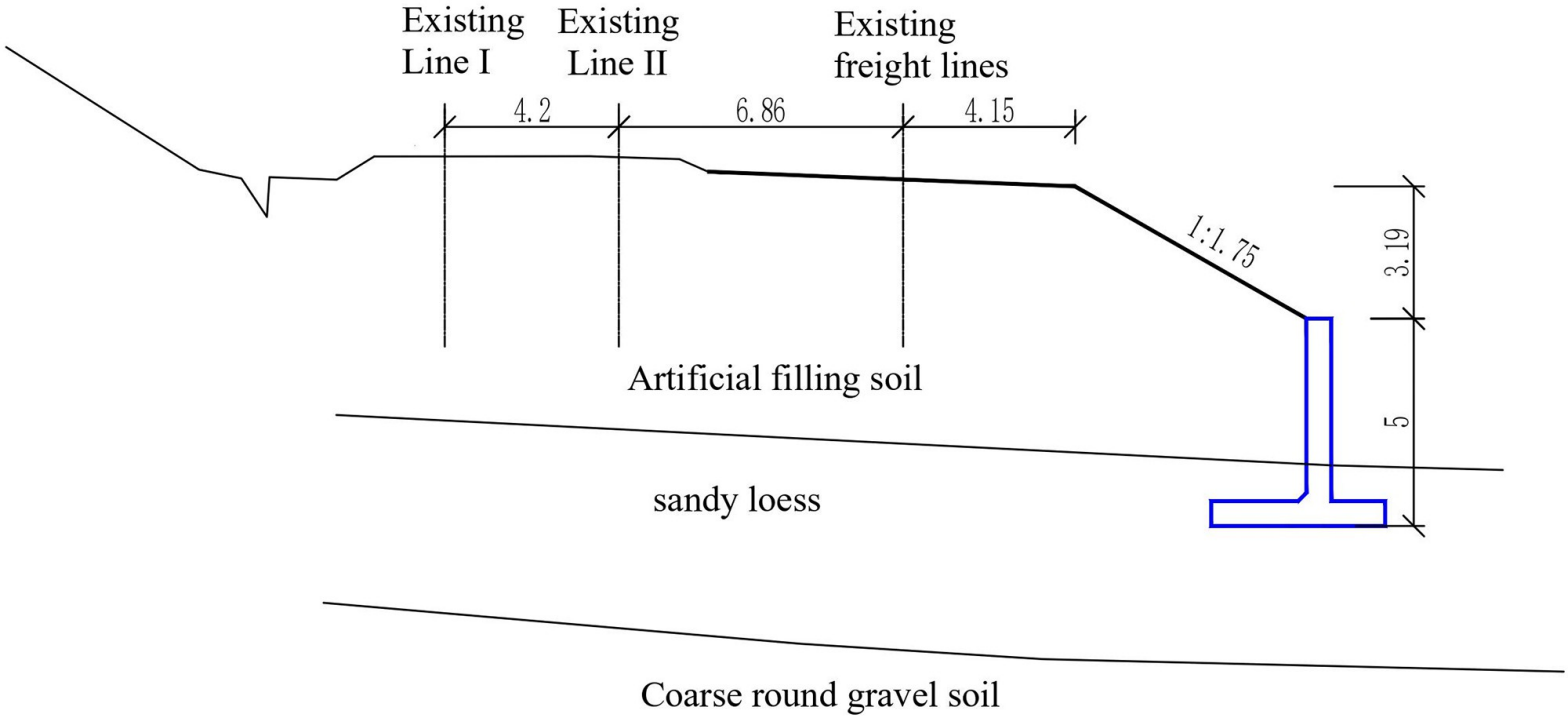
Design section of existing cantilever retaining wall (unit:m).

**Fig 2:**
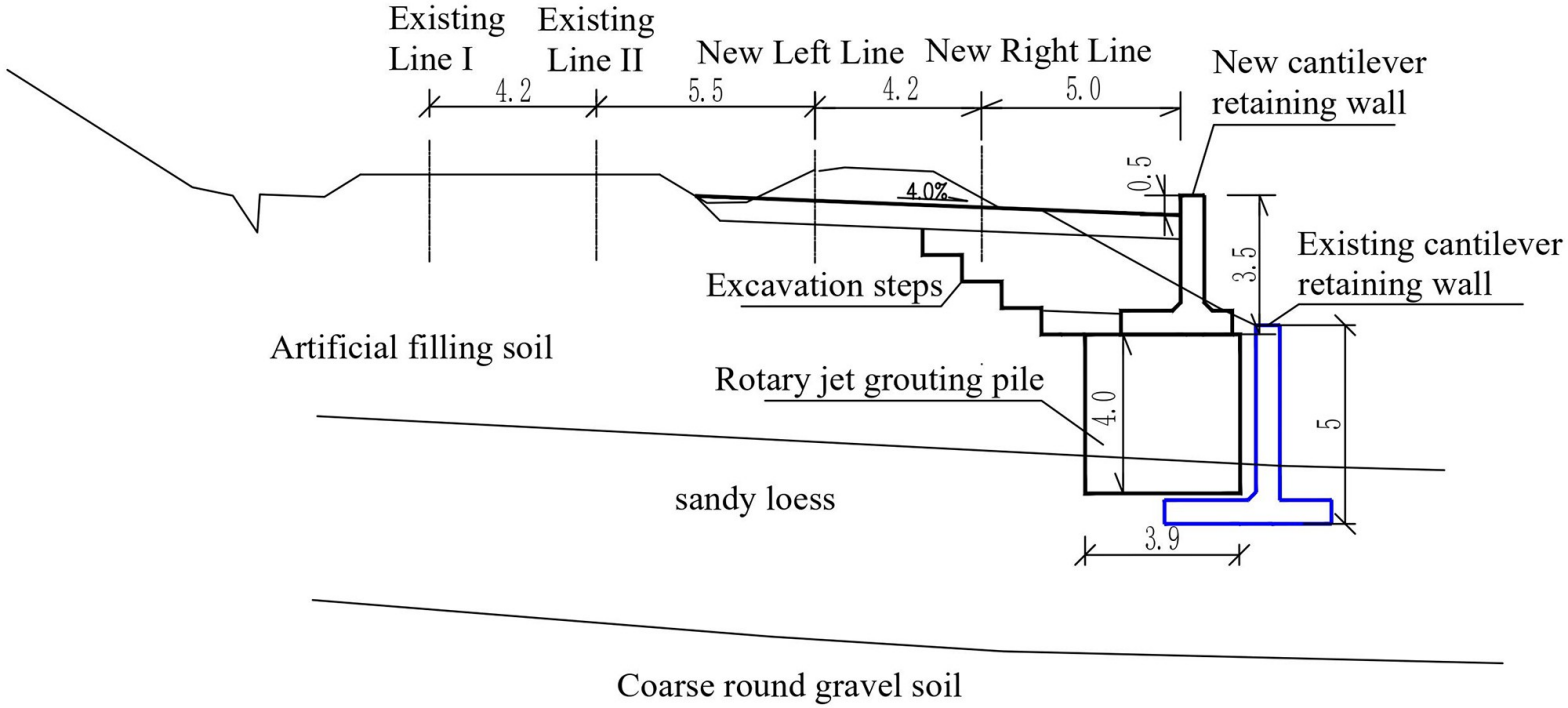
Design section of existing-new two-stage stacked cantilever retaining wall (unit:m).

This section is a supporting project for the introduction of the Zhongwei-Lanzhou passenger dedicated line to Lanzhou Hub. Site conditions for subgrade engineering are limited. The south side is adjacent to the existing Lanzhou-Xinjiang line, and the north side is adjacent to the Xijin West Road, which is the main road of the city. The existing freight line embankment was sloped by a cantilever retaining wall, as shown in Fig 1. The left line of the new Zhongwei-Lanzhou Railway needs to occupy the position of the original freight line and add the right line on the right side. Owing to the limited width of the land, to avoid affecting the Xijin West Road, it is necessary to set up a new cantilever retaining wall within the scope of the existing retaining project and then widen the subgrade on the existing embankment slope, as shown in Fig 2.

The height of the existing cantilever retaining wall is 5.0m, the segment length is 10m, the thickness of the vertical plate is 0.6m, the slope gradient above the wall top is 1:1.75, the cantilever length of the wall toe is 1.3m, the thickness of the heel and end of the wall toe is 0.6m, the cantilever length of the heel of the wall is 2.3m, the thickness of the heel and end of the wall is 0.6m, and the bottom of the bottom plate is filled with coarse grained soil.

**Fig 3:**
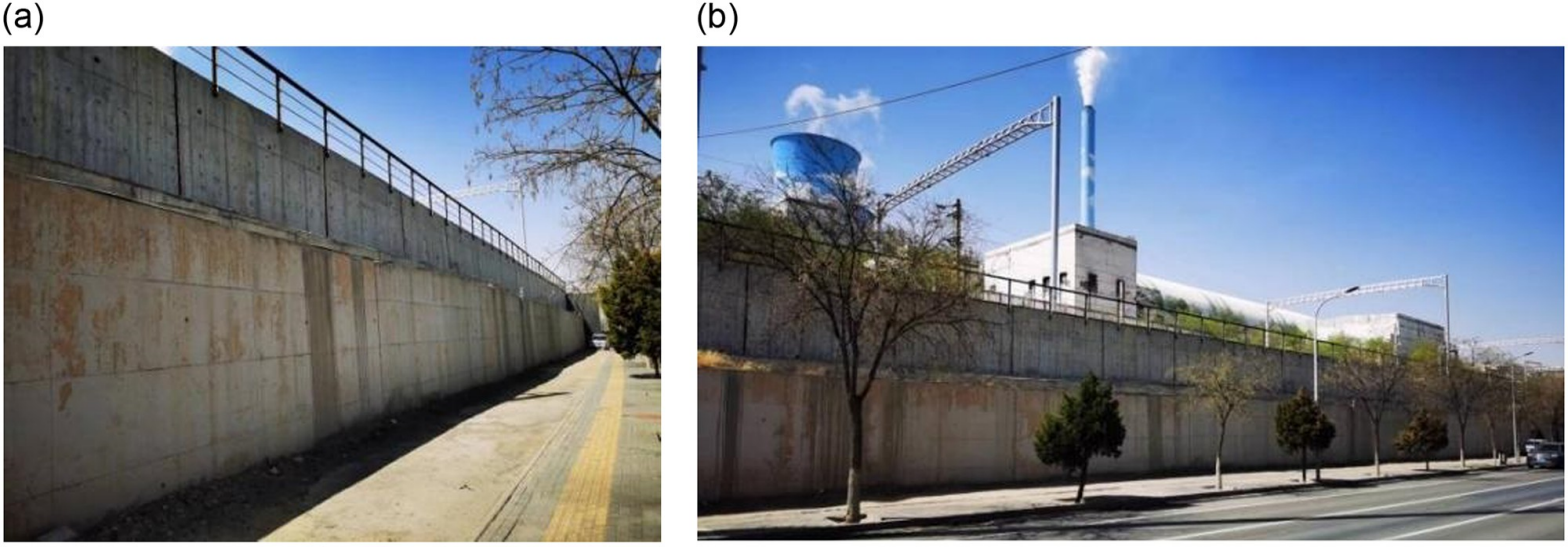
Real photo of secondary cantilever retaining wall.

The height of the new cantilever wall is 3.5m, the length of the segment is 5.0m, the thickness of the vertical plate is 0.6m, the cantilever length of the wall toe is 0.7m, the cantilever length of the wall heel is 1.5m, the height of the heel and end of the heel plate and toe plate are 0.6m, and the wall top exceeds the shoulder by 0.5m. The basement of the cantilever wall is reinforced using a jet grouting pile. The pile spacing is 1.1m, the pile diameter is 0.6m, the square arrangement is arranged, the treatment width is 3.9m, the depth is 4.0m, and a layer of 10 cm thick C25 concrete cushion is poured between the pile top and the bottom plate for leveling, and the gravel cushion is no longer set. A photograph of the scene is shown in Fig 3.

### 2.2 Engineering geological condition

The physical and mechanical parameters of each stratum according to the field investigation and geological exploration data are shown in Table 1. The geological engineering characteristics of the soil layer are as follows.

1. Artificial fill soil ($Q_{4}^{ml}$): This is distributed on the surface of existing railway embankments and highways. The height of the existing railway embankment is approximately 7~ 8m. The core was scattered, slightly dense, medium-dense, slightly wet, and grade II ordinary soil.
2. Sand loess ($Q_{4}^{al3}$): Locally distributed below the artificial filling soil layer, with a thickness of 0~6m. The composition was mainly silt, slightly dense, grade II ordinary soil, moist, σ_0_ = 120kPa.
3. Coarse round gravel soil ($Q_{4}^{al6}$):The sandy loess layer was layered, with an overall thickness of more than 40m. The composition is mainly sandstone, granite, quartzite, etc., and the rest is sand-filling, wet-saturated, dense, and grade IV soft stone, σ_0_ = 550kPa.

**Table 1:** The physical and mechanical parameters of soil layer.

stratum lithologic	γ/kN∙m^-3^	modulus of compression /MPa	poisson ratio	cohesion/kPa	internal friction angle /°
Artificial fill soil	20.1	80	0.25	5	34.5
Sand loess	17.8	10	0.28	15.0	23.2
Coarse round gravel soil	19.7	50	0.30	6.5	33.5

### 2.3 Hydrogeological characteristics

During the investigation, the aquifer was mainly a coarse, round gravel soil layer. The groundwater depth was 9~15m, which was mainly recharged by atmospheric precipitation and surface water in the deep ditch on the west side of the work site, and the water was abundant. The change in groundwater level is affected by the season and Yellow River water level, and the overall change range is generally 2~3m.

### 2.4 Monitoring section layout

**Fig 4:**
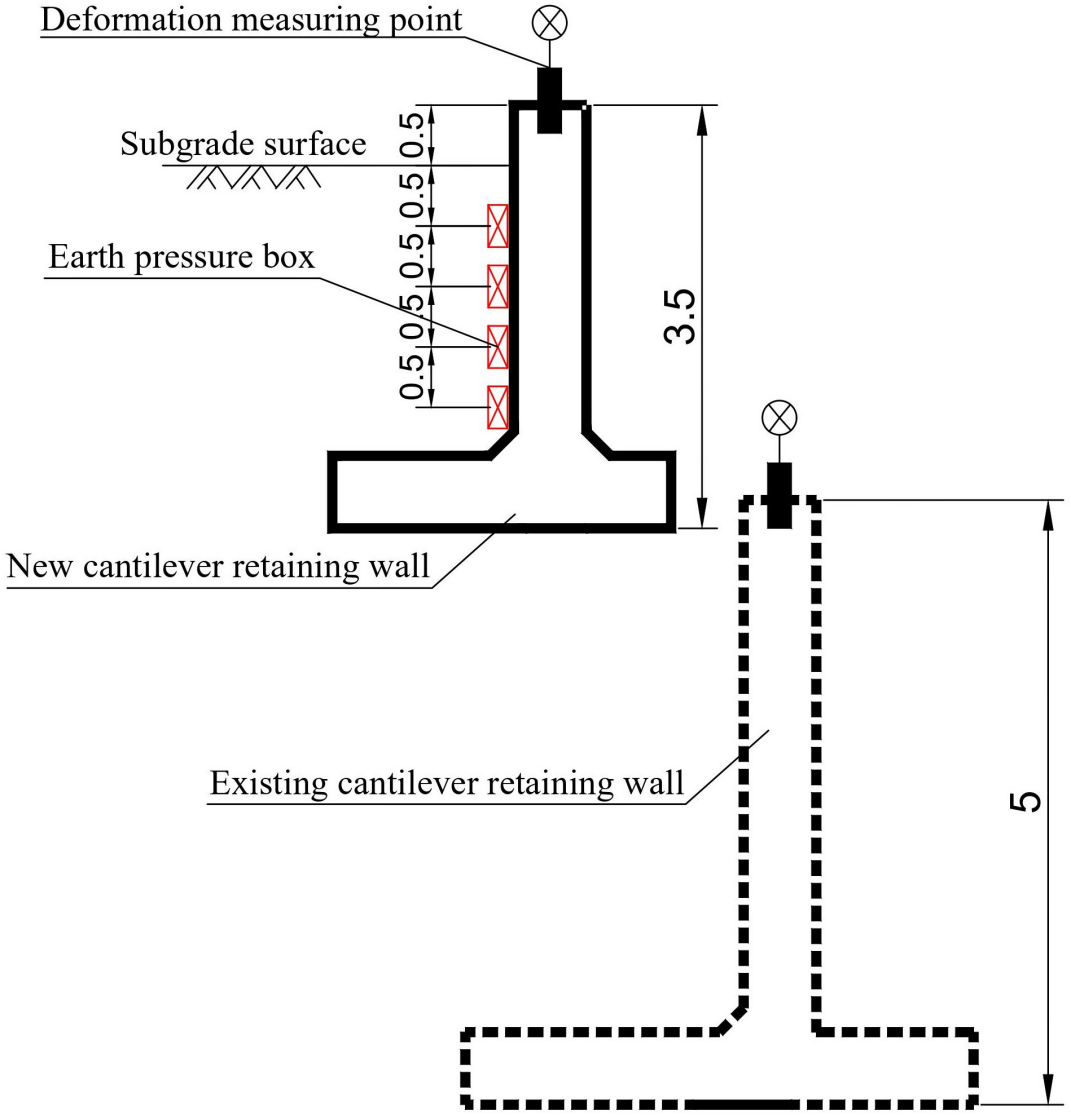
Soil pressure and deformation measuring point layout (unit: m).

To test the soil pressure on the back of the cantilever retaining wall and the deformation of the wall top during the backfill process, a typical section was selected on-site. The vibrating string double-mode earth pressure box is buried behind the new cantilever retaining wall, and the vertical spacing is 0.5 m. Displacement measuring points are arranged on the top of the new and existing cantilever retaining walls, as shown in Fig 4. According to the on-site filling situation, five test conditions were determined: the height of the subgrade filling in condition one was 1.5m. The filling height of the second working condition was 2.0 m. The filling height of working condition 3 was 2.5 m. The fourth working condition was applied to the surface layer of the subgrade bed, and the total filling height was 3m. The filling height of the fifth working condition remained unchanged, and the test time was one month later than that of the fourth working condition. The start time of the deformation test was the completion of the upper-wall concrete pouring.

## 3. Analysis of monitoring results

### 3.1 Horizontal displacement of wall top

**Fig 5:**
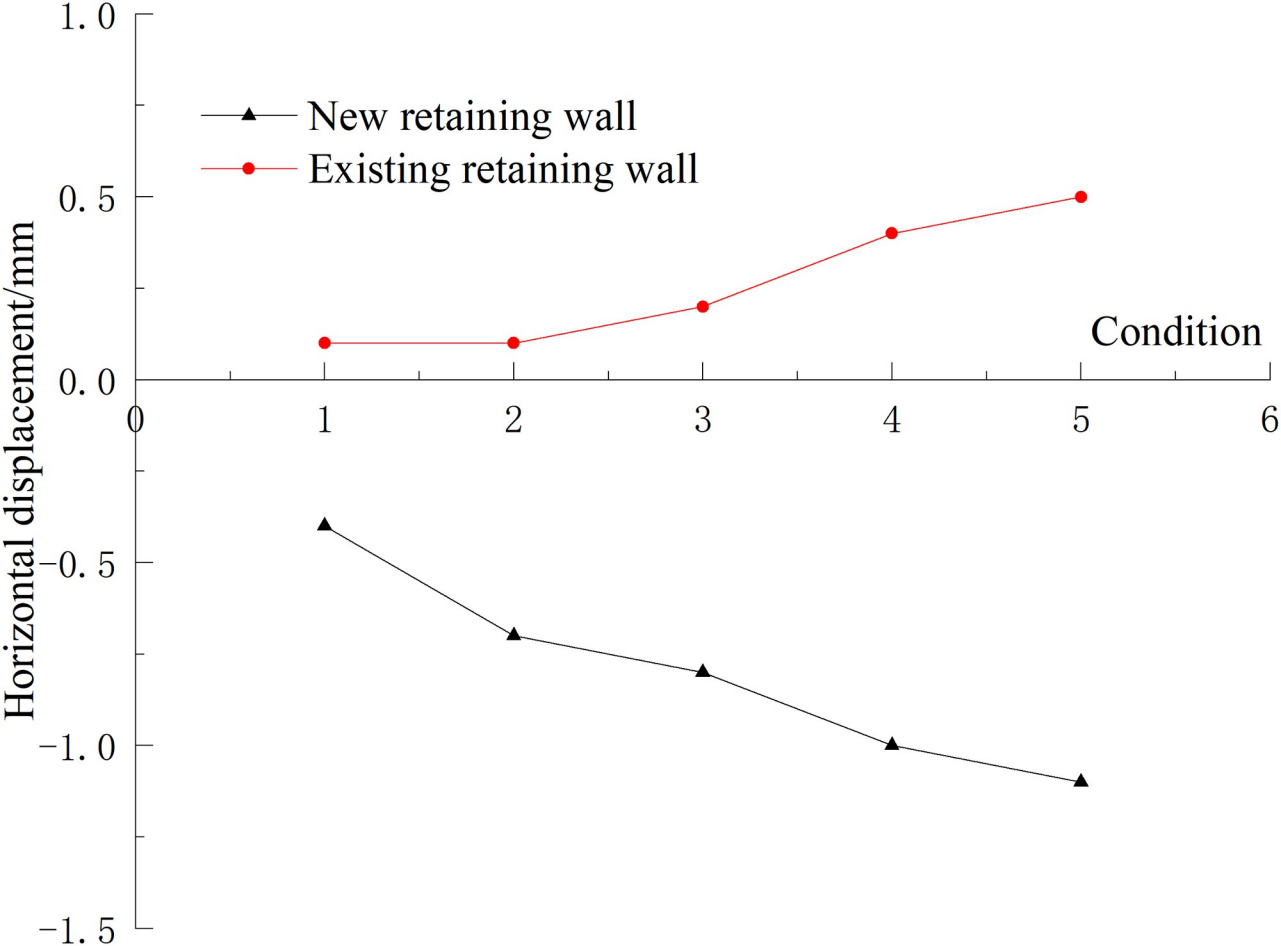
Horizontal displacement of cantilever wall top.

Fig 5 shows the horizontal displacement curves at the top of the upper and lower walls under different filling conditions. It can be seen that with an increase in the filling height behind the upper wall, the horizontal displacement of the top of the upper and lower walls increased nonlinearly. The horizontal displacement direction of the top of the upper wall was toward the filling direction, and the displacement direction of the top of the lower wall was away from the filling direction; the larger the filling height, the greater the displacement. In the filling stage, the horizontal displacement increment of the top of the upper wall is greater than the horizontal displacement increment of the top of the lower wall under the same working conditions. The main reason is that the strength and stiffness of the backfill behind the wall significantly increase after the backfill of the lower wall is reinforced by the high-pressure jet grouting pile. Most of the upper filling load was directly transmitted to the heel plate of the lower wall through the reinforced pile body. On one hand, it reduces the earth pressure acting on the lower wall. However, this increased the anti-slip stability of the lower wall. When the surface of the subgrade bed is filled, the displacement of the top of the upper wall is -1.0 mm, and the displacement of the top of the lower wall is 0.4 mm. The test results of condition 5 were slightly larger than those of condition 4 owing to the consolidation of the soil.

### 3.2 Distribution law of lateral earth pressure

**Fig 6:**
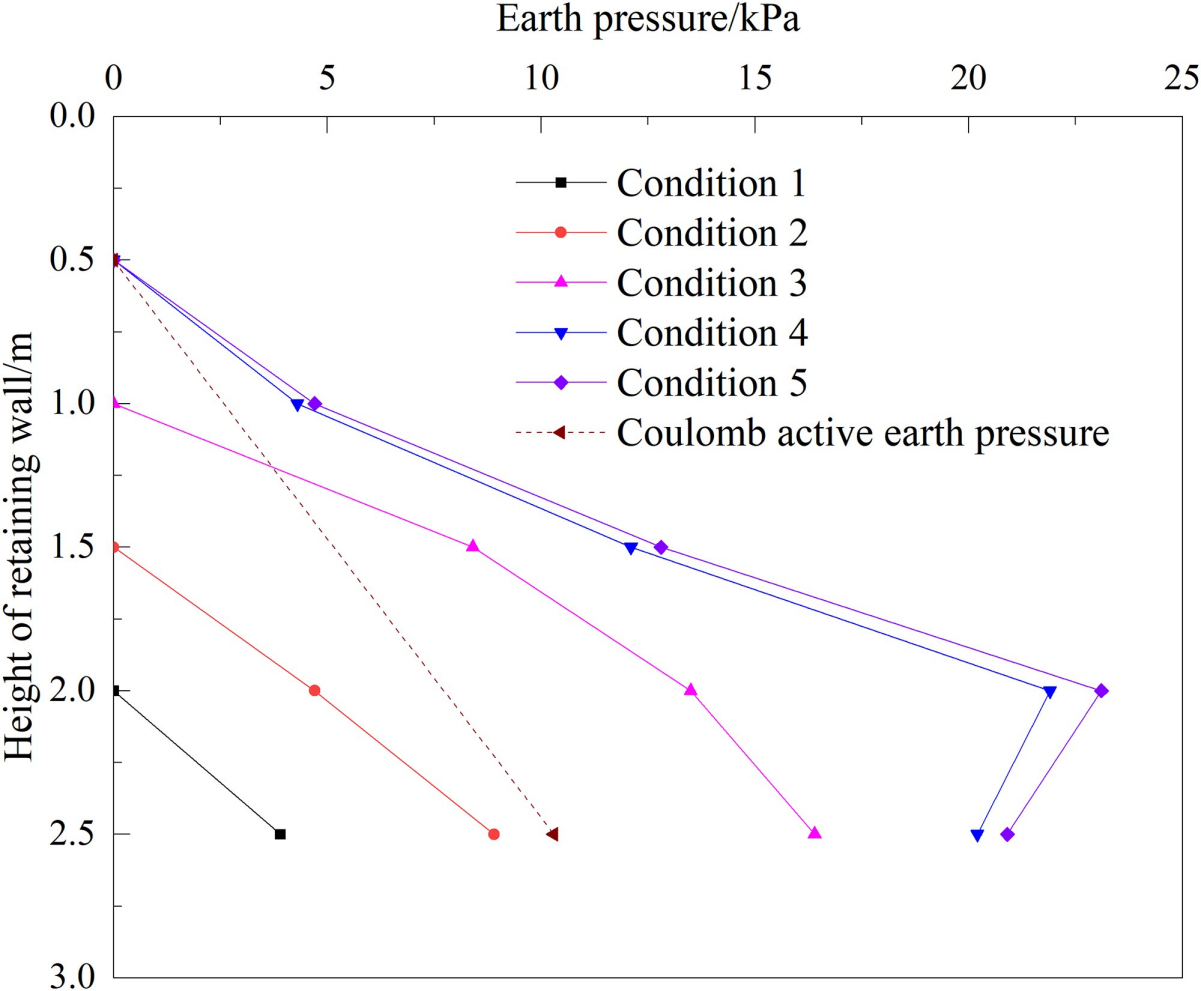
Distribution of earth pressure on the back of new cantilever wall.

Fig 6 shows the distribution of the lateral earth pressure on the back of the new cantilever wall under different working conditions. It can be seen from the figure that the earth pressure behind the wall gradually increases with an increase in the height of the filling behind the wall. The overall distribution first increases and then decreases from the surface of the backfill to the bottom of the wall and shows the characteristics of a nonlinear distribution. When the backfill behind the wall was filled to the surface of the subgrade bed, the distribution strength of the earth pressure on the back of the wall gradually increases from zero to 2 m, with a maximum value of 21.9kPa. Subsequently, it decreases slightly to the bottom of the wall, and the value is larger than Coulomb active earth pressure. The reason is that the wall has a displacement to the filling soil, and the wall squeezes the soil; thus, the earth pressure is larger. The value of condition 5 was slightly larger than that of condition 4, and the change rule was the same as that of condition 4.

## 4. Numerical calculation model and result analysis

### 4.1 Establishment of finite element model

**Fig 7:**
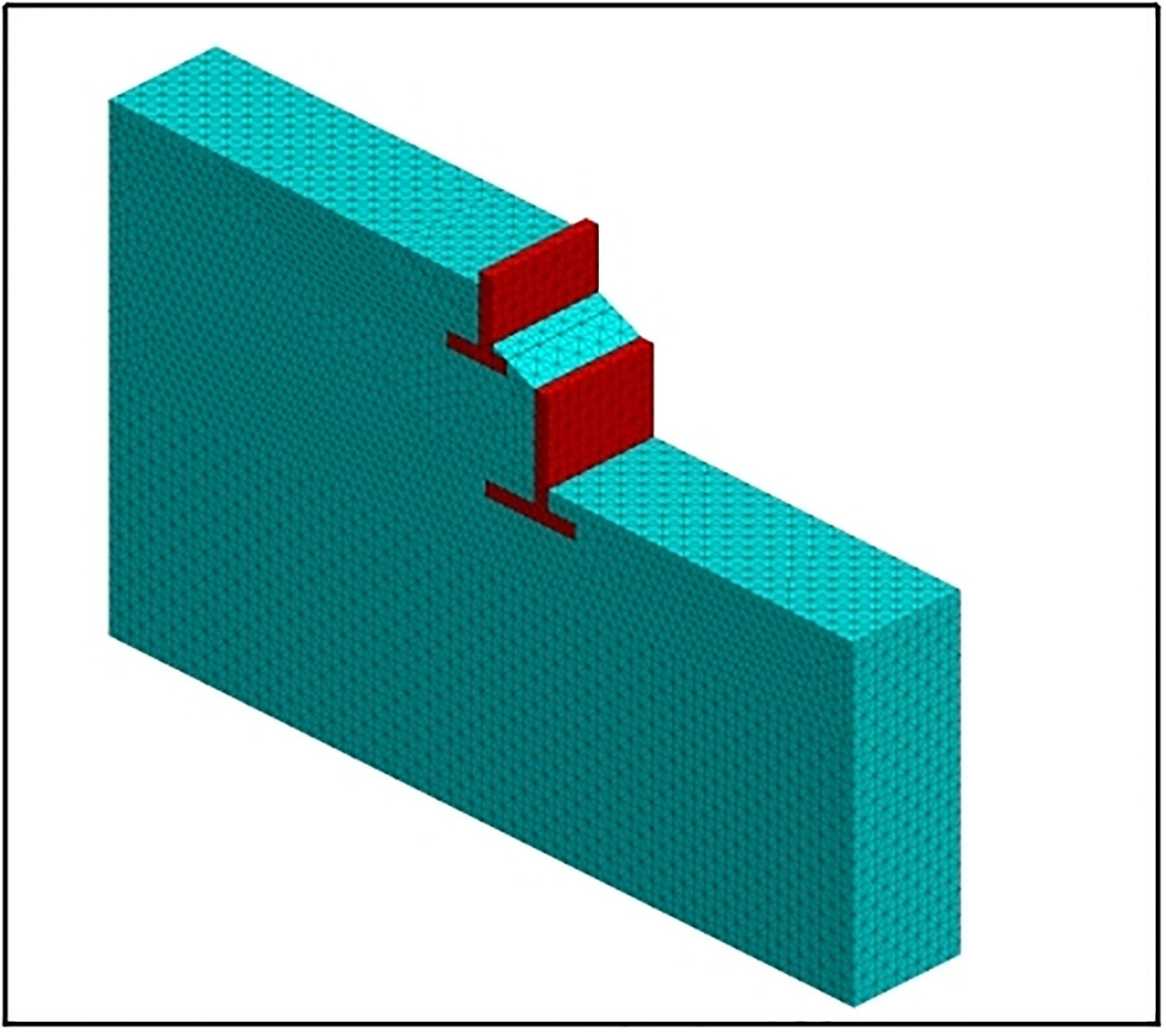
Three-dimensional model of two-stage cantilever retaining wall.

Fig 7 shows the three-dimensional finite element model of the two-stage cantilever retaining wall. The size of the model was 38m×5m×26.9m, and there were three soil layers from top to bottom. The Mohr-Coulomb ideal elastic-plastic model is used for the foundation soil and artificial filling material, and the retaining wall is a reinforced concrete structure that can be considered as a linear elastic material. The boundary conditions are fixed at the bottom and horizontal displacement constraints on both sides and the front and rear directions. To realize contact between the retaining wall and soil, the contact surface element is established. The parameters of the contact surface element are calculated according to formula (1), and k_n_ = k_s_ = 2.88E9 Pa is calculated. The calculation parameters for each soil layer are listed in Table 1, and the other calculation parameters are listed in Table 2.

$$k_{n}=k_{s}=10\;max\left[\frac{\left(K+\frac{4}{3}G\right)}{\Delta z_{min}}\right]$$ (1)

$$G=\frac{E}{2\left(1+\mu \right)}$$ (2)

$$K=\frac{E}{3\left(1-2\mu \right)}$$ (3)

where k_n_ and k_s_ are the normal stiffness and tangential stiffness of the contact surface, respectively; K is the bulk modulus; and G is the shear modulus. Δz_min_ is the minimum size of the connection area in the normal direction to the contact surface. E is the elastic modulus and μ is Poisson’s ratio.

**Table 2:** The physical and mechanical parameters of soil layer.

Stratum Lithologic	Gravity γ/ *kN∙m*^*-3*^	Modulus of Compression E /*MPa*	Poisson ratio	Cohesion /*kPa*	Internal friction angle /°
Reinforcement area of jet grouting pile	20.5	120	0.25	25	35
cantilever retaining wall	25	31500	0.2	/	/

### 4.2 Calculated work condition

For consistency with the actual construction process, the simulated calculation conditions are listed in Table 3.

**Table 3:** The calculation conditions of numerical simulation.

Working Condition	Description	Working Condition	Description
1	Simulation of existing cantilever wall	6	Filling new embankment 1.2m
2	Apply existing freight line load	7	Filling new embankment 1.8m
3	Demolition of existing freight lines and excavation steps on existing slopes	8	Filling new embankment 2.4m
4	Construction of jet grouting pile and construction of new retaining wall	9	Filling to the surface of the subgrade bed, the total filling height is 3m
5	Filling new embankment 0.6m	10	Apply track and passenger train load

### 4.3 Selection of train load

The track and train loads were selected according to the ’ Code for Design of Railway Subgrade ’ (TB10006-2016) [30]. The total load of the train and track was 68.40 kN/m^2^ under the action of the existing freight line before the embankment was widened. After the embankment is widened, the train load is 36.8 kN/m^2^, the track load is 17.3 kN/m^2^, and the line load is 10.7 kN/m^2^.

### 4.4 Calculation result analysis

#### 4.4.1 Horizontal displacement of the wall

**Fig 8:**
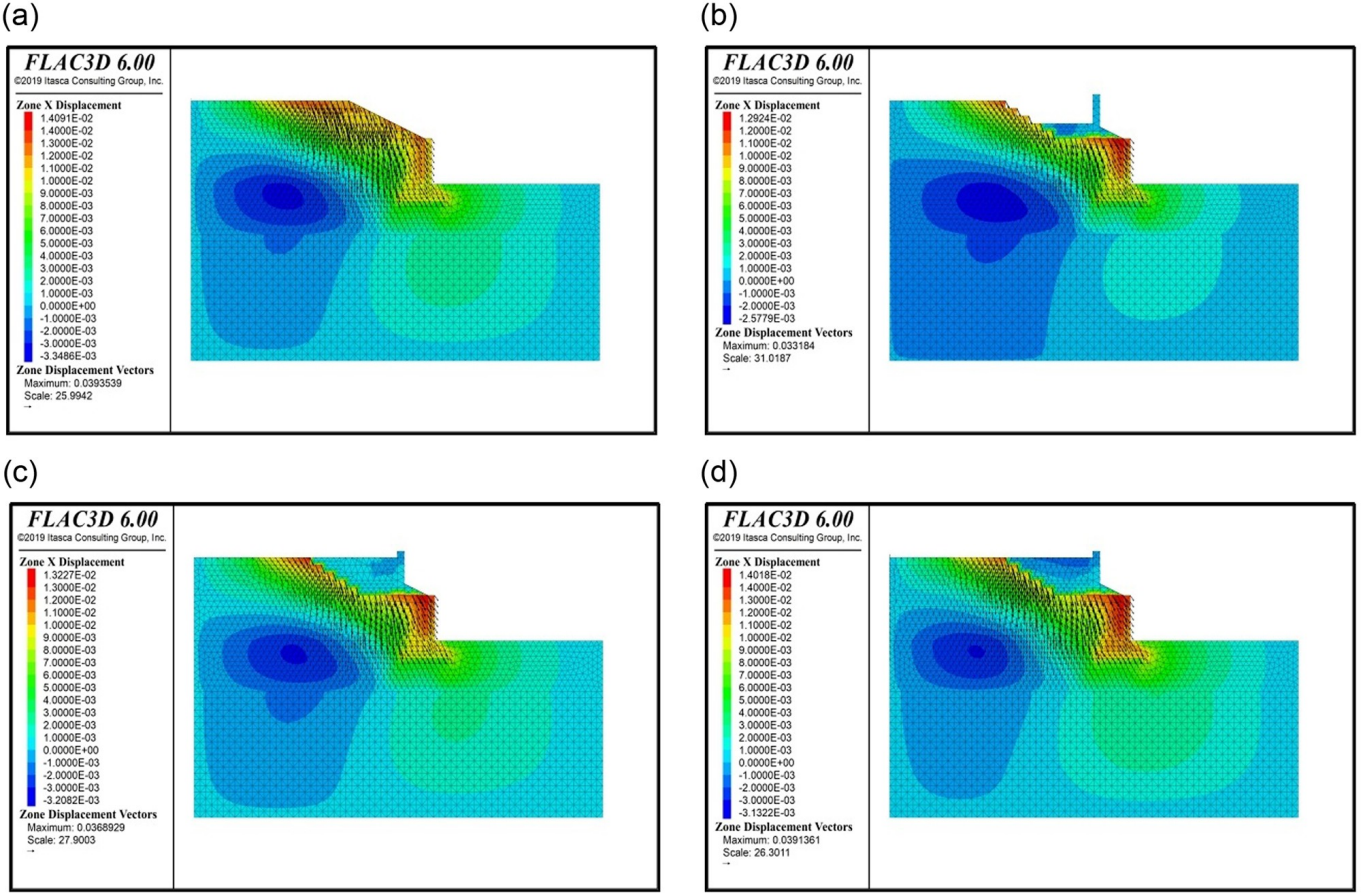
Horizontal displacement nephogram and vector diagram of typical working conditions, (a)Condition 2, (b)Condition 6, (c)Condition 9, (d)Condition 10.

Fig 8 shows the horizontal displacement cloud diagram and displacement vector diagram of the two-stage stacked cantilever wall and soil behind the wall under typical working conditions. As shown in the figure, before the embankment is widened, the existing cantilever wall undergoes horizontal displacement away from the fill and rotation around the wall toe under the action of the soil filling and train load. After the embankment was widened, with an increase in the filling height behind the new retaining wall, the displacement of the existing cantilever retaining wall gradually increased, and the horizontal displacement of the new cantilever retaining wall towards the filling direction and the rotation around the wall heel gradually increased. After the new track and train loads were applied, the displacement of the upper and lower walls increased significantly, and the displacement of the bottom of the wall increased more than that of the top of the wall. Compared with the working condition of filling the surface layer of the subgrade bed, the horizontal displacement of the bottom and top of the upper wall increased by 202% and 138%, respectively, and the horizontal displacement of the bottom and top of the lower wall increased by 20.2% and 7.7%, respectively; in other words, the inclination of the upper wall increased and the lower wall decreased.

**Fig 9:**
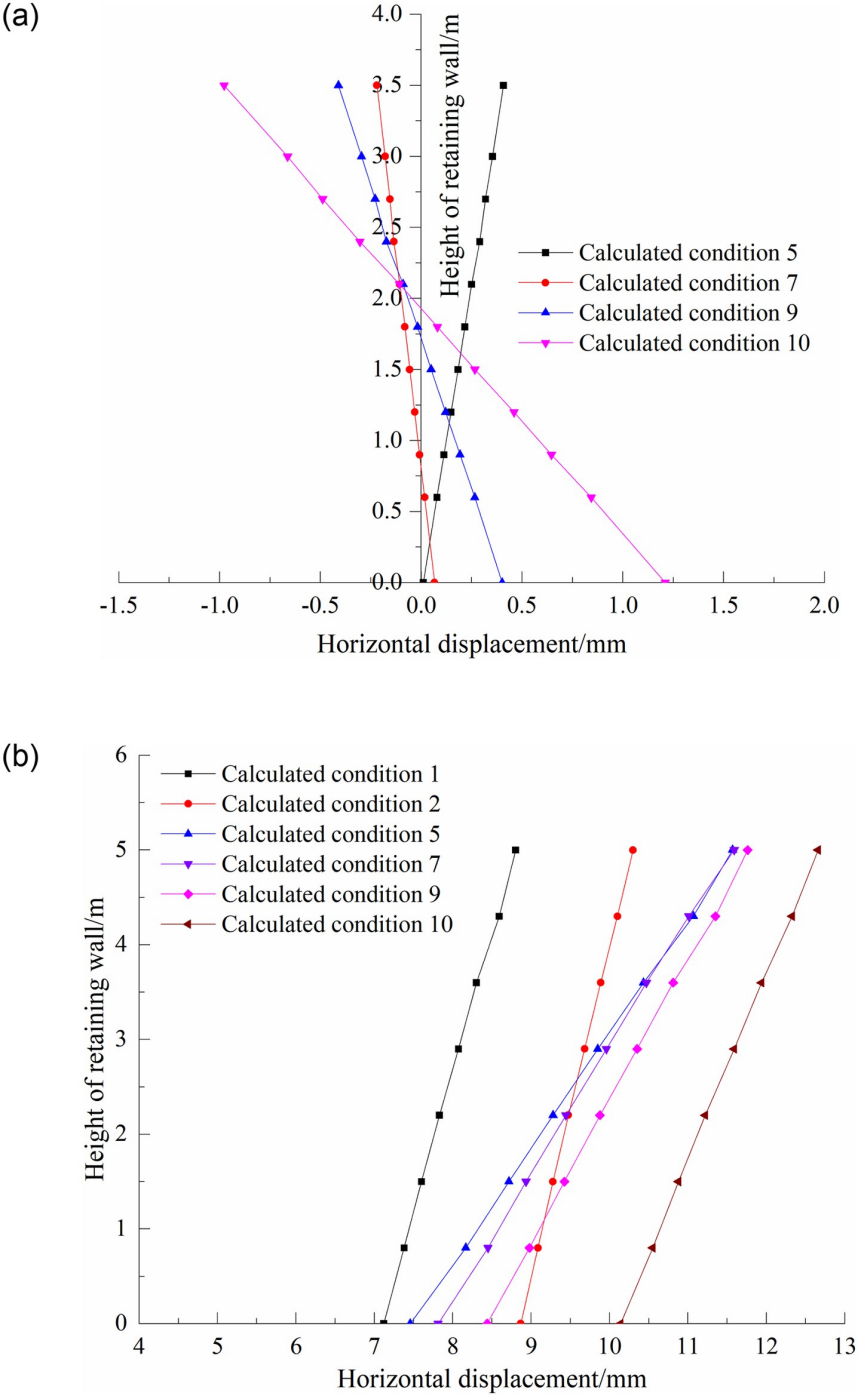
Horizontal displacement curve of wall body, (a)The upper wall, (b)The lower wall.

**Fig 10:**
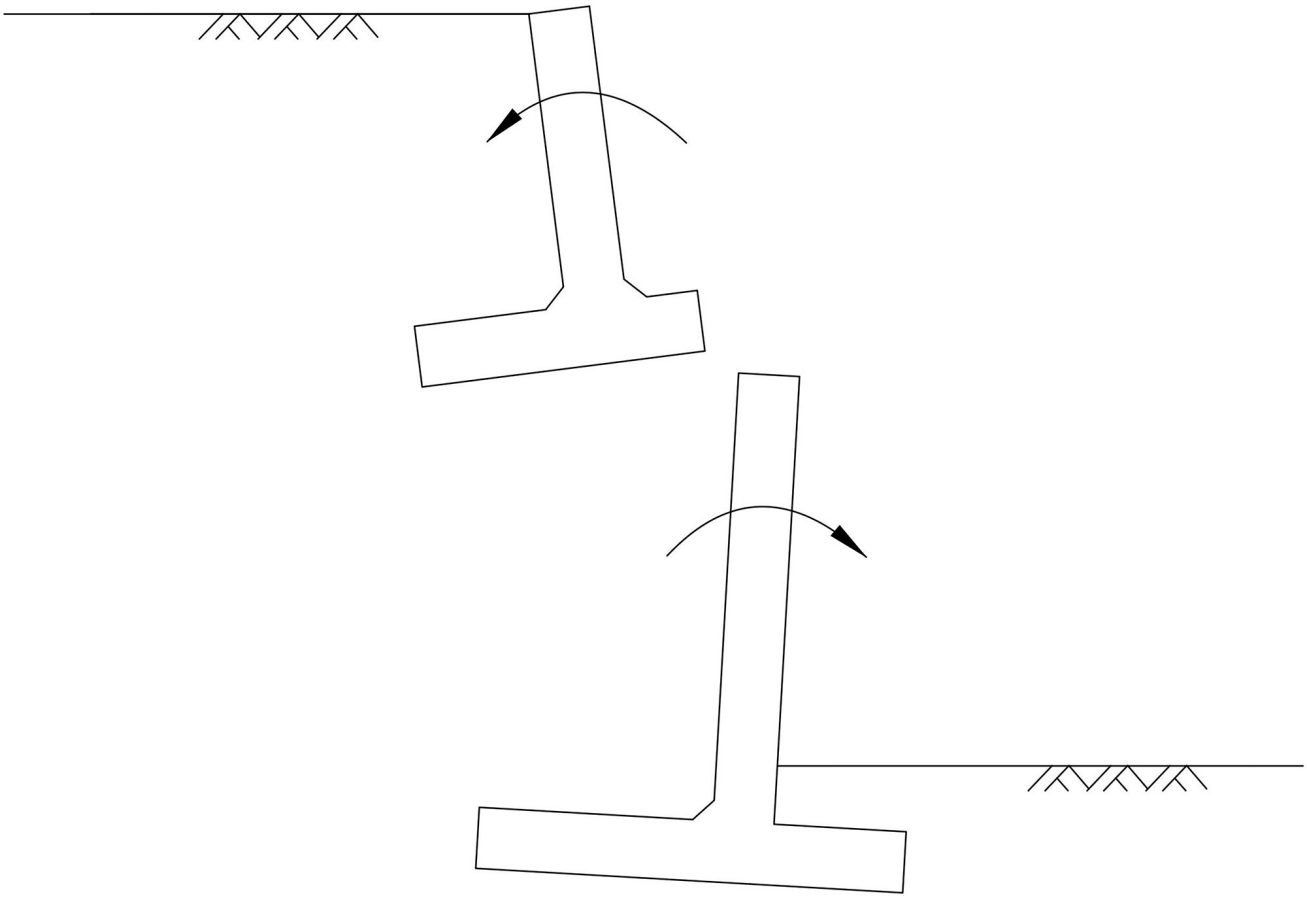
Motion trend diagram of two-stage cantilever retaining wall. 4.4.2. Earth pressure distribution.

Fig 9 shows the distribution of the horizontal displacement of the upper and lower walls along the wall height under different working conditions. As shown in the figure, the displacement of the lower wall deviates from the direction of the filling soil under the action of the existing embankment filling and freight line load, and the horizontal displacement at the top of the wall is greater than that at the bottom of the wall. Under the action of the existing embankment filling, the horizontal displacement of the top of the lower wall was 8.8 mm, and the horizontal displacement of the bottom of the wall was 7.1 mm. After the freight line train load is applied, the horizontal displacement of the wall increases by about 1.5~1.7 mm, and the displacement increment of the bottom of the wall is slightly larger than that of the wall top. After the upper wall was built and the embankment was filled, with an increase in the filling height of the embankment, the lower wall moved outward and rotated around the toe of the wall, the horizontal displacement increment of the top of the wall was obviously larger than that of the bottom of the wall, and the inclination degree of the wall body gradually increased with the increase in the filling height. When the subgrade was filled on the surface of the subgrade bed, the horizontal displacement of the top of the lower wall was 11.8 mm, and the horizontal displacement of the bottom of the wall was 8.4 mm. After applying the new line track and train load, the lower wall moves outward as a whole, the added value of the horizontal displacement at the bottom of the wall is significantly greater than that at the top of the wall, and the inclination of the wall body slows down. The overall performance of the upper wall deformation is that with an increase in the filling load, the wall first moves horizontally outward and rotates around the toe of the wall, and the horizontal displacement of the top of the wall is larger than that of the bottom of the wall; then, the top of the wall moves inward, the bottom of the wall moves outward, and the angle of the wall body moving inward becomes larger. When the new line track and train load were applied, the horizontal displacement of the top of the wall is -0.98 mm, and the bottom of the wall was 0.84 mm. This value is relatively small, mainly because the bottom plate of the upper wall is pressed on the composite foundation of the jet grouting pile, which has a large bearing capacity and overall stiffness. It can be seen that the movement of the upper wall is not only related to the magnitude of the load but also to the compactness of the filling under the bottom plate. In summary, because there is no strong connection between the upper and lower walls, under the action of load, the two show different movement trends, as shown in Fig 10, which are determined by the deformation of the soil and the force of the retaining wall.

**Fig 11:**
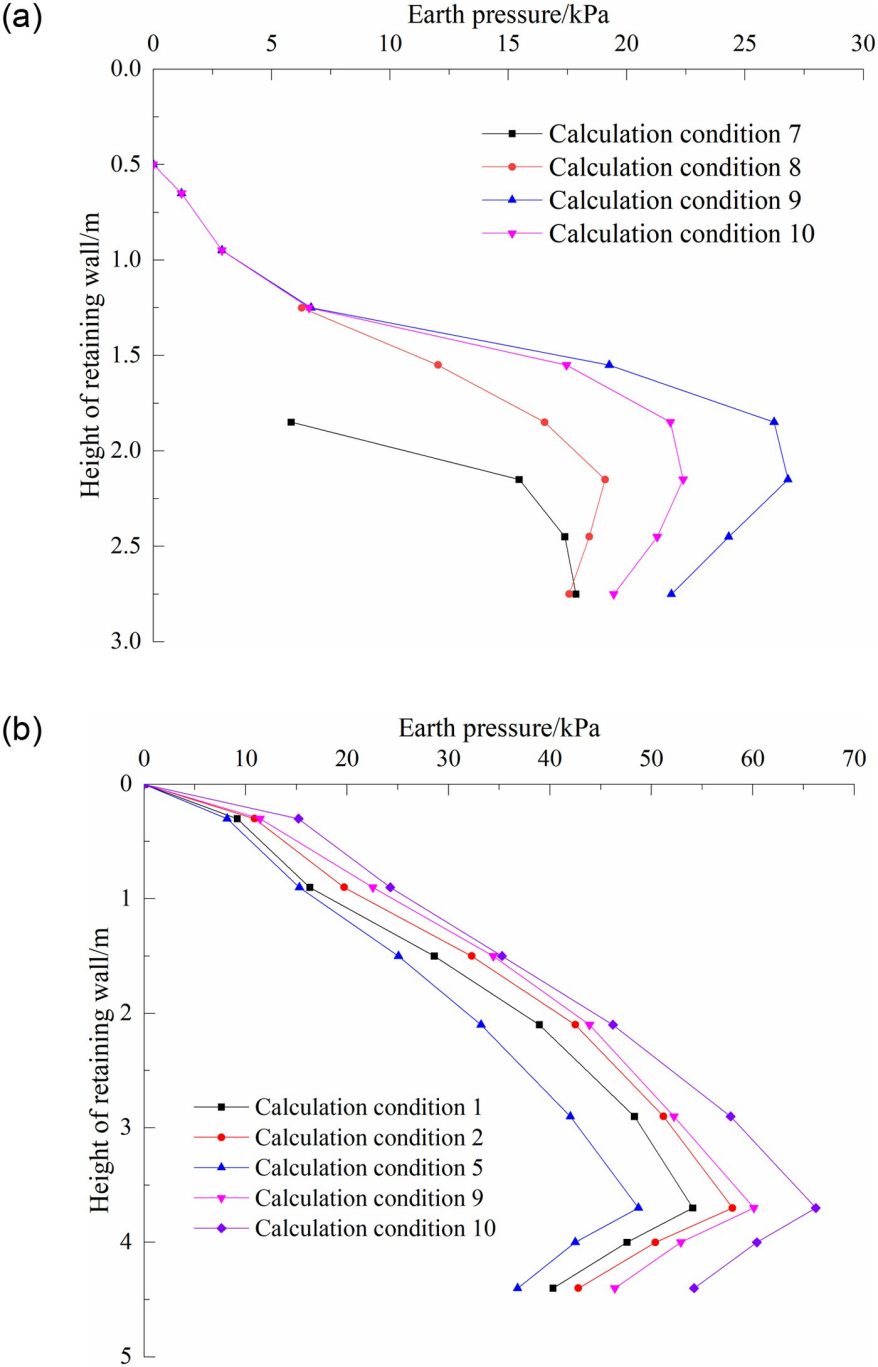
Earth pressure of two-stage cantilever retaining wall, (a)The upper wall, (b)The Lower wall.

The earth pressure calculation is the core component of the retaining structure design. Combined with engineering examples, the variation law of earth pressure of the upper and lower walls under a filling load and track train load was analyzed. Fig 11 shows the distribution of the earth pressure on the wall of the two-stage cantilever retaining wall under different working conditions. It can be seen that the variation trend of earth pressure on the lower wall under different working conditions is similar, which first increases from the top of the wall, reaches a maximum at approximately 3.7m below the top of the wall, and then begins to decrease. The maximum earth pressure distribution of the lower wall is 58.1 kPa under the action of the existing embankment fill and cargo line load. After excavating the steps on the existing embankment slope, the earth pressure on the lower wall is reduced. With an increase in the filling height behind the upper wall, the earth pressure of the lower wall increased gradually. When the embankment was filled to the surface of the subgrade bed, the maximum earth pressure of the lower wall was 60.2 kPa. After applying the new track and train load, the maximum earth pressure is 66.3kPa It can be seen that the shear strength and overall stiffness of the soil are greatly improved after the backfill of the lower wall is reinforced by the jet grouting pile, which is the main reason for the small increase of the soil pressure of the lower wall. The change trend of the earth pressure on the back of the upper wall first increased and then decreased, and the turning point occurred approximately 2.15 m below the top of the wall. It was also observed that the earth pressure on the upper wall gradually increased with increasing filling height. When the surface of the subgrade bed is filled, the maximum earth pressure on the back of the upper wall is 26.8 kPa. When the track and train loads were applied, the earth pressure on the upper wall decreased, and the maximum value was 22.4 kPa. One of the main reasons for this is that the load position is far from the back of the wall, which has little effect on the back of the upper wall. Second, the bottom of the upper wall and top of the lower wall underwent horizontal displacement away from the soil, and the earth pressure had a certain degree of unloading.

**Fig 12:**
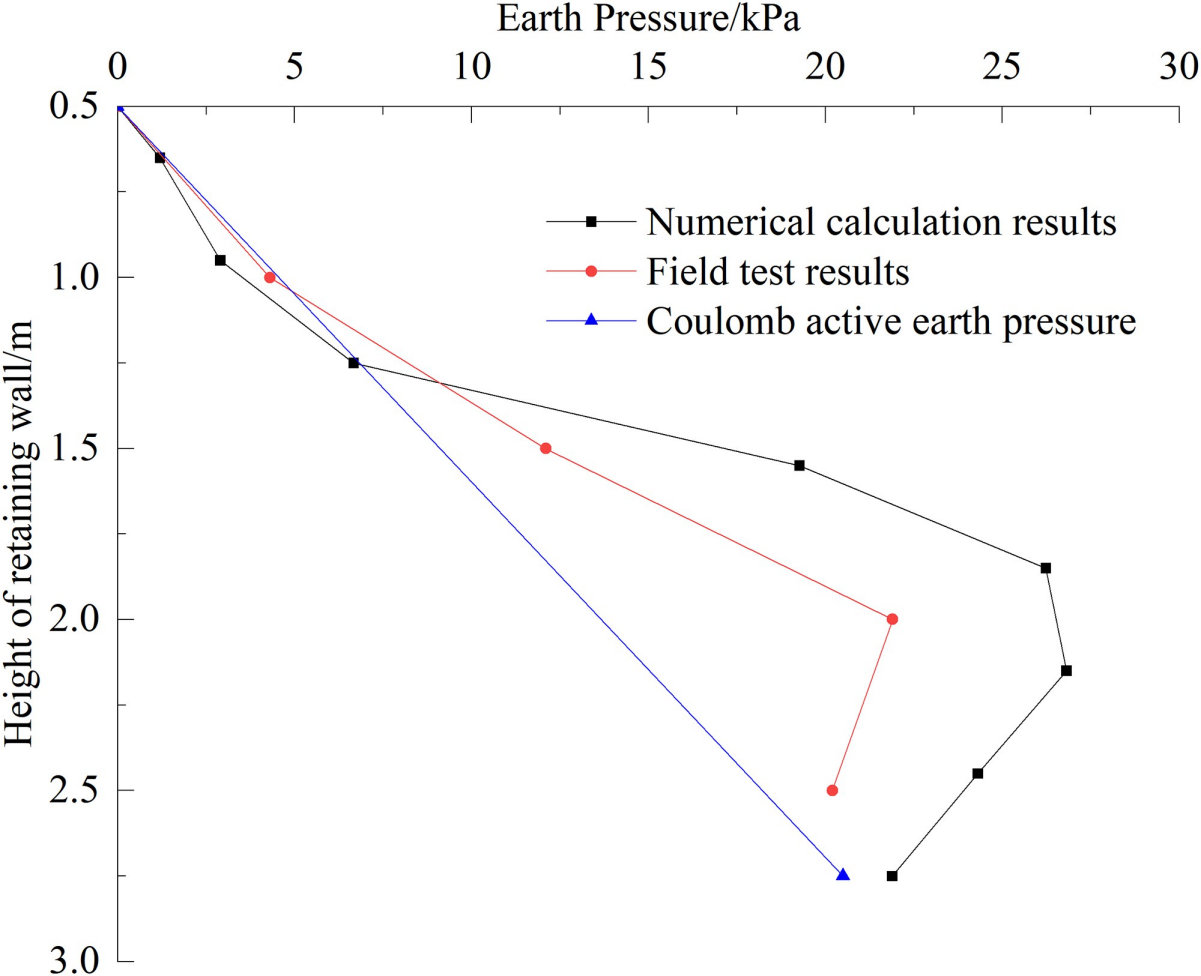
Comparison results of earth pressure.

Coulomb ’s earth pressure theory was used to calculate the active earth pressure of the upper wall when the subgrade was filled to the surface of the foundation bed, and the calculation results were compared with the field test results and numerical simulation results, as shown in Fig 12. It can be seen from the diagram that the earth pressure distribution curves obtained using different methods are different. The earth pressure calculated using Coulomb ’s earth pressure theory is the smallest. The main reason for this is that the earth pressure calculated by Coulomb ’s theory is the earth pressure under the limit equilibrium state. The results of the other two methods are the earth pressure under the non-limit state finite displacement condition, and the numerical calculation assumes that the wall back filler is a continuous medium, which is different from Coulomb ’s earth pressure theory, which assumes that the wall back soil is an ideal granular material, so the results obtained are quite different. The numerical simulation results were consistent with the field test results, the numerical deviation was not large, and the soil pressure at the bottom of the wall was reduced. The accuracy and reliability of the numerical model calculation results were verified through comparison.

#### 4.4.2 Overall stability analysis

The anti-sliding stability of the two-stage stacking retaining wall was calculated based on the strength reduction finite element method. That is, in the finite element calculation, for a certain assumed strength reduction factor F, the original strength parameters of each soil layer are reduced according to formula (4) to carry out elastic-plastic finite element calculation of the slope. According to a certain instability criterion [33], the slope reaches the limit equilibrium state and the corresponding strength reduction factor F is the anti-sliding safety factor Fs of the retaining wall. Otherwise, the newly assumed reduction factor is repeated until the soil reaches the critical limit equilibrium state.

$$c_{r}=c/F,\varphi_{r}=\text{arctan}\left(tan\varphi /F\right)$$ (4)

where *c* and *φ* are the cohesion and internal friction angles of the soil layer, respectively. *c*_r_ and *φ*_r_ are the reduced cohesion and internal friction angles, respectively.

**Fig 13:**
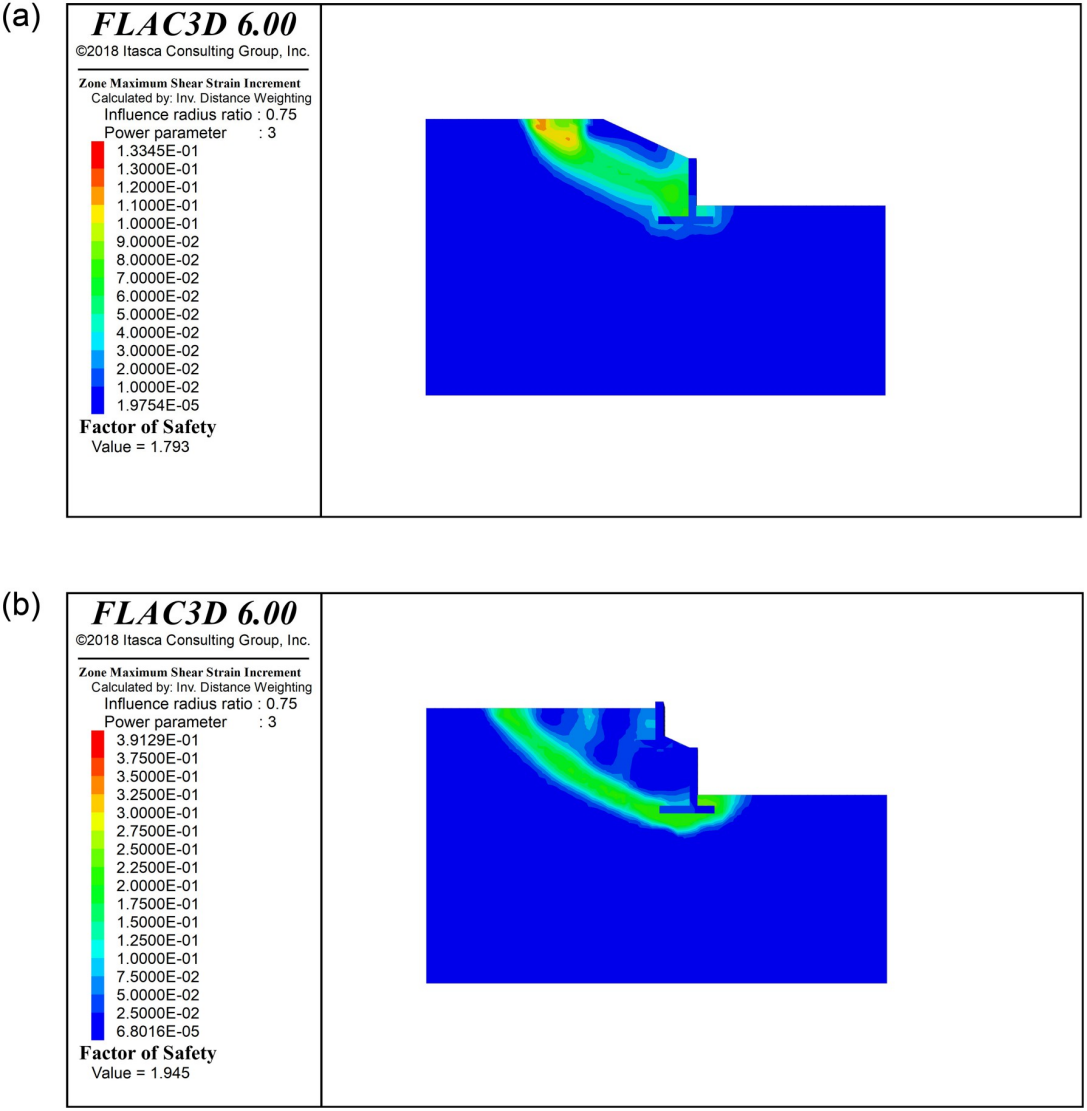
Maximum shear strain increment cloud diagram, (a)The existing cantilever retaining wall, (b) The two-step cantilever retaining wall.

Based on the strength reduction method, the potentially most dangerous sliding surface and overall stability safety factor of the retaining wall are obtained when the slope reaches the limit equilibrium state. Fig 13 shows the maximum shear strain increment cloud diagram of the existing cantilever retaining wall and two-stage cantilever retaining wall under the limit state. Whether it is a single-stage or two-stage cantilever retaining wall, the failure mode of this type of embankment slope is the overall sliding failure of the retaining wall and the soil behind the wall. The sliding surface passes through the lower edge of the wall heel of the lower wall, and cuts out from the vicinity of the wall toe. The entire sliding surface shape was similar to an arc shape. It can also be seen from the figure that the overall stability safety factor of the existing embankment cantilever retaining wall is 1.793, and the overall stability safety factor of the existing-new two-stage cantilever retaining wall is 1.945. In comparison, the stability of the two-stage cantilever retaining wall was better.

## 5. Design calculation method of existing-new two-stage cantilever retaining wall

The structural type of the two-stage cantilever retaining wall is relatively new, and its design calculation method has not yet been established. There is no corresponding design regulation in the current design code for retaining structures. Therefore, combined with the research results of this study, a calculation method for the overall stability and earth pressure of a two-stage cantilever retaining wall is proposed.

### 5.1 Global stability

**Fig 14:**
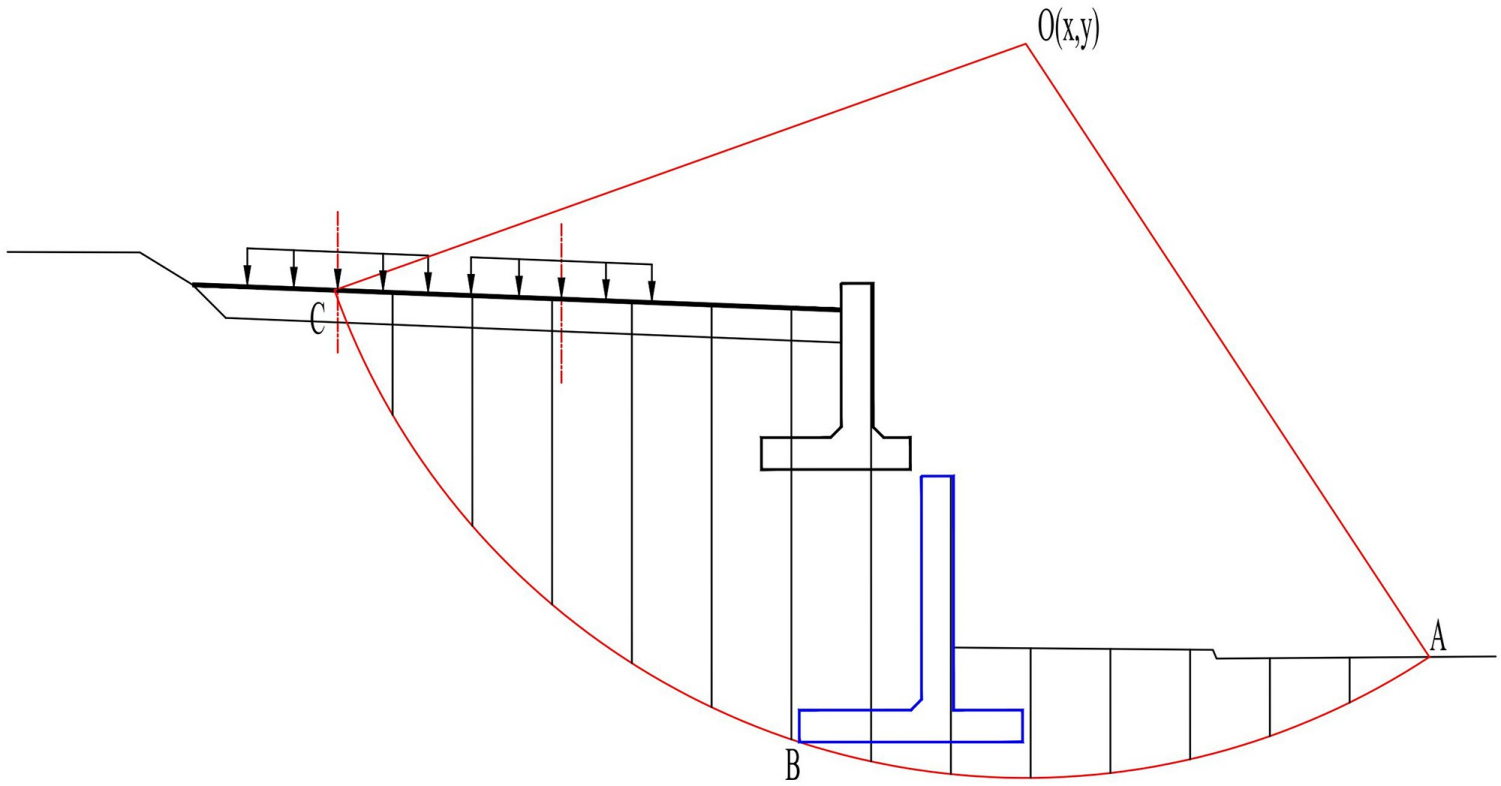
The overall stability analysis diagram of the two-stage cantilever retaining wall.

Based on the potential failure characteristics of the two-stage cantilever retaining wall embankment slope shown in Fig 13, the shape of the sliding surface can be simplified as an arc tangent to point B at the lower edge of the lower retaining wall, as shown in Fig 14. The sliding soil and two-stage cantilever retaining wall can be divided into several vertical strips (The train load and the weight of the wall are divided into the weight of the corresponding soil bar respectively), and the stability coefficient of the slope is solved using the arc sliding slice method. The stability coefficient *K* is a function of the coordinates of the center of the circular arc. Taking the stability coefficient as the objective function, the center coordinates corresponding to the minimum objective function were searched to obtain the most dangerous sliding surface position and the corresponding stability coefficient. The above algorithm can be realized using MATLAB.

### 5.2 Earth pressure

The retaining slope of the two-stage cantilever retaining wall should not only satisfy the overall stability of the slope, but also satisfy the local stability of the retaining walls at all levels, such as anti-sliding stability, anti-overturning stability, base stress, and eccentricity. The key is to determine the size and distribution of the earth pressure on retaining walls at all levels.

The active earth pressure at the back of the upper wall can be calculated using Coulomb ’s earth pressure theory. In the calculation, it was necessary to determine whether the second fracture surface appeared in the filling soil. If the inclination angle of the imaginary wall connecting the upper edge of the vertical plate and the lower edge of the wall heel is greater than the critical angle, the earth pressure should be calculated according to the theory of the second fracture surface.

**Fig 15:**
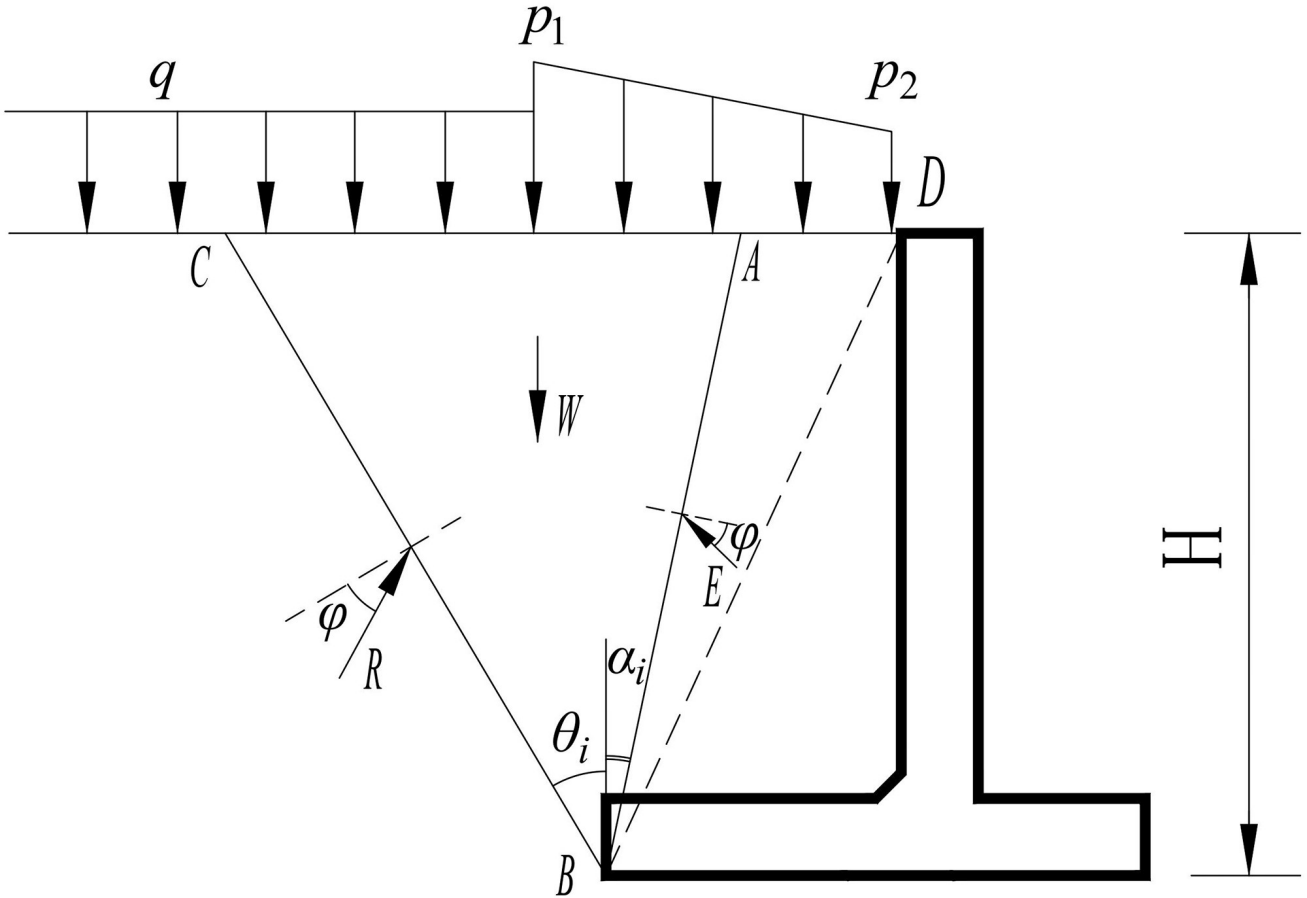
Calculation mode of active earth pressure of lower wall.

When calculating the earth pressure on the lower wall, the effect of the upper wall is simplified as the external load acting on the top of the lower wall. Because the base pressure of the upper wall is generally non-uniformly distributed under the action of earth pressure, wall weight, and self-weight of the filling between the imaginary wall back (or the second fracture surface) and retaining wall, the linear distribution model can be considered for a simplified analysis. In addition, the self-weight of the fill above the top of the lower wall and the load of the track train should be simplified to the plane of the top of the lower wall, and the active earth pressure of the lower wall should be calculated according to the Coulomb earth pressure theory. If a second fracture surface appears, the earth pressure should be calculated according to the second fracture surface theory, as shown in Fig 15. Among them, the inclination angle of the first slip surface BC is *θ*_*i*_, and the inclination angle of the second slip surface BA is *α*_*i*_ = 45°-φ/2. The distributed loads of the CD section on the filling surface can be simplified as the equivalent load *p* and *q*. The gravity *W* of the fractured soil wedge ABC and the resultant force *Q* of the distributed load on the filling surface in the range of the sliding wedge are related to the fracture angle *θ*_*i*_ of the sliding surface. According to the static equilibrium condition of the slip soil wedge ABC, the earth pressure *E* on the second rupture surface can be obtained:

$$E_{a}=\left(W+Q\right)\frac{\text{sin}\left(\theta_{i}-\varphi \right)}{\text{cos}\left(\alpha_{i}+2\varphi -\theta_{i}\right)}$$ (5)

Using *dE*/*dθ*_*i*_ = 0, the fracture angle *θ*_*i*_ of the sliding surface is obtained and substituted into Eq (5), the active earth pressure Ea on the second sliding surface of the lower wall can be obtained as In addition, the lower wall is an existing retaining wall, and the earth pressure generated by the existing embankment filling and freight line load on the lower wall should also be calculated. If the effect of the existing embankment excavation step on the earth pressure of the lower wall is ignored, the larger earth pressure of the lower wall in the above two cases is considered as the design load.

## 6. Conclusion

Based on the actual project, a new cantilever retaining wall is built on the slope of the existing embankment cantilever retaining wall for widening the roadbed. The soil pressure, deformation and overall stability of the new cantilever wall and the existing cantilever wall during the construction of the new cantilever retaining wall and the filling of the roadbed were studied by means of field monitoring and numerical simulation. The main conclusions are as follows:

With the increase of the filling height behind the upper wall, the horizontal displacement of the top of the upper and lower walls increases nonlinearly. The displacement direction of the top of the upper wall is the direction of filling, and the displacement direction of the top of the lower wall is the direction of deviating from the filling. The larger the filling height is, the larger the displacement increment is, and the horizontal displacement increment of the top of the upper wall is greater than that of the top of the lower wall.
Because there is no strong connection between the upper wall and the lower wall, the upper wall and the lower wall show different movement trends. The lower wall moves outward and rotates around the wall toe to the free surface. The horizontal displacement of the top of the wall is obviously larger than that of the bottom of the wall. The bottom of the upper wall moves outward, the top of the wall moves to the direction of filling, and the whole tilts to the direction of filling. In contrast, the displacement of the upper wall is much smaller than that of the lower wall.
With the increase of the filling height behind the wall, the earth pressure behind the upper wall gradually increases from the filling surface to the bottom of the wall, and decreases slightly to the bottom of the wall, but the overall distribution is approximately linear. After the filling soil behind the lower wall is reinforced by the jet grouting pile, the change trend of the earth pressure behind the wall under all working conditions is basically similar. It increases first from the top of the wall, reaches the maximum value at about 3.7m below the top of the wall, and then begins to decrease, and the change range of the earth pressure is small.
Whether it is a single-stage or two-stage cantilever retaining wall, the failure mode of the embankment slope is the overall sliding failure of the retaining wall together with the fill soil. The sliding surface is cut out from the vicinity of the wall toe through the lower edge of the wall heel of the lower wall, and the whole sliding surface shape is similar to the arc shape.
(5) Based on the field measurement and numerical simulation results, a calculation method for the overall stability and earth pressure of the existing two-stage cantilever retaining wall was proposed.
